# Changes in Sexuality, Body Image and Health Related Quality of Life in Patients Treated for Hematologic Malignancies: A Longitudinal Study

**DOI:** 10.1007/s11195-016-9459-3

**Published:** 2016-10-15

**Authors:** Cecilia Olsson, Ann-Kristin Sandin-Bojö, Kaisa Bjuresäter, Maria Larsson

**Affiliations:** Department of Health Sciences, Faculty of Health, Science and Technology, Karlstad University, Karlstad, Sweden

**Keywords:** Body image, Chemoimmunotherapy, Health related quality of life, Hematologic malignancies, Longitudinal study, Sexuality

## Abstract

Longitudinal studies exploring sexuality, body image and health-related quality of life (HRQoL) are lacking in patients treated with chemo- or chemoimmuno-therapy for hematologic malignancies. The aim was to describe and explore changes in sexuality, body image and HRQoL in patients treated for hematologic malignancies, from baseline until 6 months after treatment. Twenty patients above 45 years (median age 62) treated for DLBCL, CLL or AML participated. Data were collected at baseline, 1- and 6-months after treatment by means of three instruments: SAQ-S, BIS and EORTC QLQ-C30. The results showed that patients’ sexuality was negatively affected 1 month after treatment, but after 6 months the patient reported scores had returned almost entirely to baseline scores. Body image was slight negatively affected after 1 month and after 6 months, 50 % reported that body image was not affected at all. Regarding HRQoL, patients reported gradually improved scores during the study period. Regression analysis showed that changes in sexuality and body image seemed to influence changes in HRQoL. This study has shown changes in sexuality, body image and HRQoL over time in patients above age 45 treated for hematologic malignancies. One month after treatment all three areas becomes negatively affected, and thereafter the patients’ scores recovered to a great extent regarding these issues within 6 months. Sexuality and body image seem to be important aspects of HRQoL for these patients and need to be integrated in the cancer rehabilitation during and after treatment.

## Background

In Sweden, about 1400 individuals (median age 70 years) are diagnosed with diffuse large B cell lymphoma (DLBCL), chronic lymphocytic leukemia (CLL) or acute myeloid leukemia (AML) every year [1]. Recent developments within the field of supportive care have enabled the treatment of patients who previously were considered to be too fragile to manage the expected side-effects [2]. Hence, the number of patients being treated and surviving the diseases have increased during the last years. Cancer diagnosis and associated treatments often imply complex and long-lasting physical and psychosocial problems for the individual [2, 3]. Therefore, there is an increasing need to focus on health related quality of life (HRQoL) issues both during and after treatment. Research in patients treated for various hematologic malignancies shows that HRQoL is most negatively affected at diagnosis, during treatment [3–5] and at the initial period after treatment. Thereafter, physical, psychological and emotional well-being gradually improves [3, 5]. However, the knowledge is scarce regarding in what ways and for how long sexuality is affected in patients treated for hematologic malignancies.

Sexuality in nursing research is described as a concept that includes physiological, psychological, social and socio-cultural dimensions interacting in a complex manner [6–10]. It is acknowledged that cancer diagnosis and associated treatments have an impact on all these dimensions in a multifaceted way [9–12]. In spite of this, studies focusing sexuality in non sex-specific cancer populations are lacking. The existing research focusing on sexuality in patients with hematologic malignancies mostly concerns fertility [13] and/or sexual function [14–18] in rather young patients, treated for Hodgkin lymphoma [13–16], or patients who have undergone hematopoietic stem cells transplantation [17, 18]. These studies show that in general patients experience affected sexuality during treatment and for a short period afterward. Furthermore, being female [10], older age [13, 19], in an advanced state of illness, having poorer physical health and high degree of emotional distress [19, 20], seem to be associated with a higher risk for affected sexuality such as reduced sexual function [13–18], and decreased sexual desire [21]. In addition, a qualitative study has shown that both women and men diagnosed with DLBCL, CLL and AML, experienced negative effects on their sexual relationships during and after treatment [22]. However, research in patients over the age of 45 treated for hematologic malignancies and longitudinal studies that examine sexuality in relation to HRQoL in this special group of patients is very rare [4].

Body image is one important aspect of both sexuality and HRQoL, which have been studied to a very limited extent in patients with hematologic malignancies [22, 23]. However, Weber et al. [23] found that body image was negatively affected in newly diagnosed patients and Olsson et al. [22] have shown that both women and men experience changes in appearance leading to feelings of being sexually unattractive, decreased sexual desire and avoidance of intimacy.

In summary, the issue of how sexuality is affected in patients with hematologic malignancies is examined to a limited extent and previous studies have mainly focused on fertility and function in rather young patients or in patients treated with hematopoietic stem cells transplantation. However, patients with hematologic malignancies such as DLBCL, CLL and AML are most often in their 50s or older and the majority is treated with chemo- or chemoimmuno-therapy. Furthermore, longitudinal studies and studies that examine sexuality from a multidimensional perspective, including sexual interest, sexual satisfaction and sexual function in relation to HRQoL and body image in patients with hematologic malignancies are rare. Studies such as these are needed as these could give important information to be used when developing adequate supportive care programs for this special group of patients.

## Aim

The aim was to describe and explore changes in sexuality, body image and HRQoL in patients treated for hematologic malignancies, from baseline until 6 months after treatment.

## Methods

### Study Design and Data Collection and Sample

This longitudinal study present data from three occasions for measurement: after the second cycle of chemo- or chemoimmuno-therapy (Baseline), 1 month-(Follow-up 1) and 6 months after completion of treatment (Follow-up 2). Data was collected between June 2010 and January 2013 at seven units in four hospitals in central Sweden. Patients were included consecutively. The study has a descriptive and explorative design. Inclusion criteria were patients with one of the diagnoses DLBCL, AML or CLL and treated with chemotherapy or chemoimmunotherapy (i.e. in this study a combination of chemotherapy agents and monoclonal antibodies). An additional criterion used was that the patients should be ≥45 year and able to read and write Swedish. Exclusion criteria were earlier treatment for hematologic malignancies, relapsed disease or stem cells transplantation. The Swedish Cancer Registry at the Regional Cancer Centre Uppsala-Örebro was used in order to identify potential participants. Registered nurses responsible for carrying out patients’ treatment and care served as patient recruiting nurses. In connection with the second cycle of treatment, the patient recruiting nurses gave verbal information about the study and asked the patients verbally about participation. Written information about the aim and design of the study, voluntariness and confidentiality was also given to the patients. When patients agreed to participate, the patients’ demographic and medical characteristics were collected by the recruiting nurses, and the researcher (CO) sent the first package of questionnaires to the patient by ordinary mail. The second package (Follow-up 1) was sent 1 month after the treatment was completed and the third package (Follow-up 2) was sent 6 month after the treatment was completed. Up to two reminders were sent, 2 weeks apart.

**Fig. 1:**
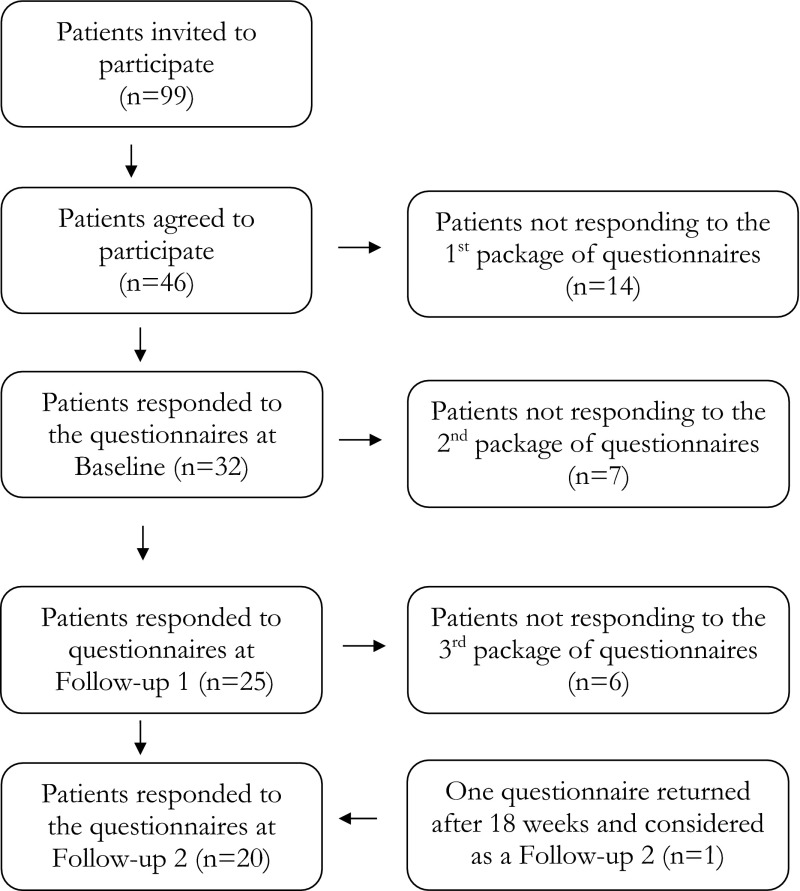
Flow chart for sample and drop-outs for the study

Initially 99 patients were invited to participate and 46 of those agreed. However, 12 men and two women never returned the questionnaires at baseline (mean age 67 years, range 52–78). Out of the 32 responding patients at baseline, 25 patients also responded at ‘Follow-up 1’, 1 month after the treatment was completed. Data for the 32 patients at baseline and 25 patients at Follow-up 1 is presented in an earlier study [24]. At ‘Follow-up 2’, 6 month after treatment, 20 patients responded the questionnaires. One woman returned the first follow-up nearly 6 month after treatment, this questionnaire was used as a ‘Follow-up 2’ instead, which is shown in the flow chart of number of responding and non-responding participants (Fig. 1).

The known reason for not participate in this longitudinal study was due to; patient considered the content of the instrument as irrelevant at the present time of life (n = 4), comorbidity (n = 1), relapse of disease (n = 3) or death (n = 2). There were no statistically significant differences regarding sex, age, civil status, level of education and comorbidity between the responding and the non-responding group of patients at baseline, Follow-up 1 and Follow-up 2.

### Instrument

#### The Sexual Adjustment Questionnaire-Swedish version

*The Sexual Adjustment Questionnaire* (SAQ) is an instrument developed to measure changes in sexuality (over time) in cancer patients, and has undergone validity and reliability testing [25, 26]. The SAQ [19] was translated into Swedish (SAQ-S) by the research team. The translation procedure is described in detail earlier [24] and additionally further down in the paragraph Body Image Scale (BIS). Permission to translate and use the SAQ was obtained from the author of the original instrument.

SAQ-S exists in four versions, one baseline and one follow-up, for men and women respectively. The SAQ-S consists of 16 unisex items at baseline, of which fifteen had a corresponding item in the follow-up version. One question is only in the baseline version and two occurs only in the follow-up version. The baseline versions measure ‘how sexuality was experienced before diagnoses’ and the follow-up version measure ‘how sexuality is experienced at present’. There are also sex-specific question, one ‘female’ and two ‘male’. The response options represented a five-point Likert scale, except for one item with a six-point scale. There was also a possibility to score ‘not applicable’. High scores indicate more positive feelings or functioning in the item areas. To calculate a SAQ-S total mean value, all unisex items mean value were added and divided by the number of item. According to the constructor of SAQ, professor Wilmoth (personal contact, October 2, 2013) the items could be grouped into the three modified scales for men. On the bases of the content, they were labeled as follows: *sexual interest* (six items), *sexual function* (five items) and *sexual satisfaction* (five items). In the present study, the SAQ-S was modified to be unisex, and thus was used for both men and women. Therefore, the two gender-specific items were removed in the *Sexual Function* scale and the unisex item *did*/*do you have problems with your sexual ability* was included. In the scale *sexual satisfaction*, the item *did*/*do you feel tense or frustrated after a sexual experience* was previously removed from the Swedish version due to cross-cultural aspect during the translation. Internal consistency was calculated with Cronbach’s alpha coefficient [27], which was at the three occasion of measurement, respectively: *sexual interest*, .75, .88, .87, *sexual function* was .86, .41, .84 and *sexual satisfaction* was .73, .86, .60.

#### Body Image Scale

The *Body Image Scale* (BIS) was developed for assessment of cancer patients’ perception of their own body image and consist of 10 items which have undergone validity and reliability testing [21]. Cronbach’s alpha has earlier shown good values .78–.92 [28, 29]. BIS was translated into Swedish by the research team. Permission to translate and use the BIS was obtained from the author of the original instrument [28]. The translation procedure was done according to recommended guidelines for cross-cultural adaption [30, 31]. Two bilingual persons, one with knowledge of the subject, translated the instrument from English into Swedish. Some issues were discussed with persons with specialist knowledge of the subject, and the research team content- and cultural-validated the instrument. Thereafter, a bilingual third translator blindly translated the Swedish version into English. In this study we removed the items *have you been feeling that the treatment has left your body less whole* and *have you been dissatisfied with the appearance of your scar*, since these items were not applicable for our study group. A four point Likert like scale represented the item response options, ‘Not at all’, ‘A little’, ‘Quite a bit’ and ‘Very much’, which score 1–4, respectively. The mean value of all items were added and divided by eight in order to calculate a BIS total mean value. The BIS total score of all items in this study could vary by 8–32, with a lower score representing a better body image. Internal consistency was calculated with Cronbach’s alpha coefficient [27] which for BIS was .91 at Follow-up 1, and .92 at Follow-up 2.

#### The European Organization for Research and Treatment of Cancer Quality of Life Questionnaires

*The European Organization for Research and Treatment of Cancer Quality of Life questionnaires* (EORTC QLQ-C30, version 3) is an instrument for assessment of cancer patients HRQoL which have undergone extensive validity and reliability testing [32, 33]. Cronbach’s alpha has earlier been considered acceptable for the multi-item scales ranged .52–.89 [32]. EORTC QLQ-C30 consists of five functional scales: physically, mentally, emotionally, socially and cognitively and three symptom scales: fatigue, nausea/vomiting, and pain, five single symptoms items about shortness of breath, insomnia, loss of appetite, constipation, diarrhea, as well as one concerning Global Health status/QoL scale. The 30 item instrument also includes a single item about financial difficulties. All items cover experiences ‘during the last week’. A four point Likert like scale represented the item response options ‘Not at all’, ‘A little’, ‘Quite a bit’ and ‘Very much’. The two items in Global Health status/QoL scale have a seven-point range from ‘Very poor’ to ‘Excellent’. The scales and single-item measures from 0 to 100, where a higher functional scale score represent a high level of functioning and a high score for global health status/QoL represent a high HRQoL. On the contrary a low score on one symptom scale represent a low level of problems/symptoms [34]. Internal consistency was calculated with Cronbach’s alpha coefficient [27], which for the multi-item scales ranged from .55 to .96 at Baseline, from .48 to .93 at Follow-up 1, and from .31 to .95 at Follow-up 2.

### Ethical Approval

The research was carried out in accordance with ethical principles and guidelines as outlined in ‘Ethical guidelines for nursing research in the Nordic Countries’ [35], in line with the ‘Declaration of Helsinki’ [36]. The study was approved by the Regional Ethical Review Board in Uppsala (Dnr. 2010/065). Written informed consent was obtained when the questionnaires were completed and sent back to the researcher. Permission to carry out the study was given by the respective Head of departments from which the informants were recruited.

### Statistical Analysis

Descriptive statistics were used to describe patient data: frequencies, percent, mean, median (MD) and standard deviation (SD). Fisher’s exact test and *t* test were used to examine differences between the non-responding participants compared with the responding participants at Baseline, Follow-up 1 and Follow-up 2. Mean values for the study group was illustrated by using 95 % confidence intervals (CI) for the *SAQ*-*S total*, *Global Health status*/*QoL* and for *BIS*. For the comparison with *SAQ-S total* the scores for *Global Health status*/*QoL* was converted from 0–100 to 0–5. Wilcoxon signed-rank test was used to examine differences between related groups, at ‘Baseline–Follow-up 1’ and at ‘Follow-up 1–Follow-up 2’.

The area under study is unexplored, therefore linear regression analyses [27] were carried out to further explore how changes in differences for sexuality and body image explained changes in differences for quality of life between the occasions for measurement. The summarized individual mean values of the ‘changes in differences’ between the occasions for measurement were calculated for each scale (*diff 1* = difference between Baseline and Follow up 1: and *diff 2* = difference between Follow up 1 and Follow up 2). *Diff 1 and diff 2* was used as variables in the analysis. Dependent variable was the outcome measures *Global Health status*/*QoL Scale diff.* The independent variables *Sexual Interest diff*, *Sexual Function diff*, *Sexual Satisfaction diff*, *Body Image Scale diff* and *Sex* were entered simultaneously. Three participants were excluded list-wise in the SAQ-S in the regression analyses. All statistical tests were two-tailed, and *P* ≤ .05 was considered statistically significant [27]. All statistical analyses were performed using the Statistical Package for Social Sciences (SPSS) version 20.0.

## Results

This study is based on 20 patients (13 men and 7 women) with a mean age of 62.5 years (Md = 62.5, range 50–79 years), who remained in the study at 6 months after treatment. The majority of the participants had a partner and education at the university level. The predominant diagnosis was DLBCL, and most patients were treated with chemoimmunotherapy (R-CHOP). Patient demographics and medical characteristics are shown in Table 1. However, the number of individuals varied in the different analyzes depending on the patients’ use of the response alternative ‘not applicable’ and a few internal drop-outs. All of the patients included (n = 20) answered the SAQ-S and the EORTC QLQ-C30 at Baseline. At Follow-up 1, 19 patients answered the SAQ-S and 18 patients answered the EORTC QLQ-C30 and the BIS. At Follow up 2, all of the 20 patients answered the three instruments.

**Table 1:** Demographic and medical characteristic of participants

Characteristics	n	(%)
	(n = 20)	
*Sex*		
Men	13	(65)
Women	7	(35)
*Age*		
Mean	62.5	
Median	62.5	
45–54	2	(10)
55–64	10	(50)
65–74	6	(30)
75–84	2	(10)
*Civil status*		
Married/cohabitation	14	(70)
Living apart	2	(10)
Living alone	3	(15)
Widow/widower	1	(5)
*Education level*		
Elementary school	6	(30)
Upper secondary school	3	(15)
University	10	(50)
No answer	1	(5)
*Employment*		
Employed	10	(50)
Unemployed	1	(5)
Retired	7	(35)
On sick leave	2	(10)
*Diagnosis*		
Diffuse large B cell lymphoma (DLBCL)	14	(70)
Chronic lymphocytic leukemia (CLL)	3	(15)
Acute myeloid leukemia (AML)	3	(15)
*Chemimmunotherapy*		
R-CHOP^a^	13	(65)
Other treatment regimen^b^	7	(35)

^a^ *R*-*CHOP* rituximab, cyclophosphamide, doxorubicin, vincristine and prednisolone

^b^ *FCR* fludarabin cyclophosphamide and rituximab or *AD* arabine/cytarabine and daunarubicin

**Fig. 2:**
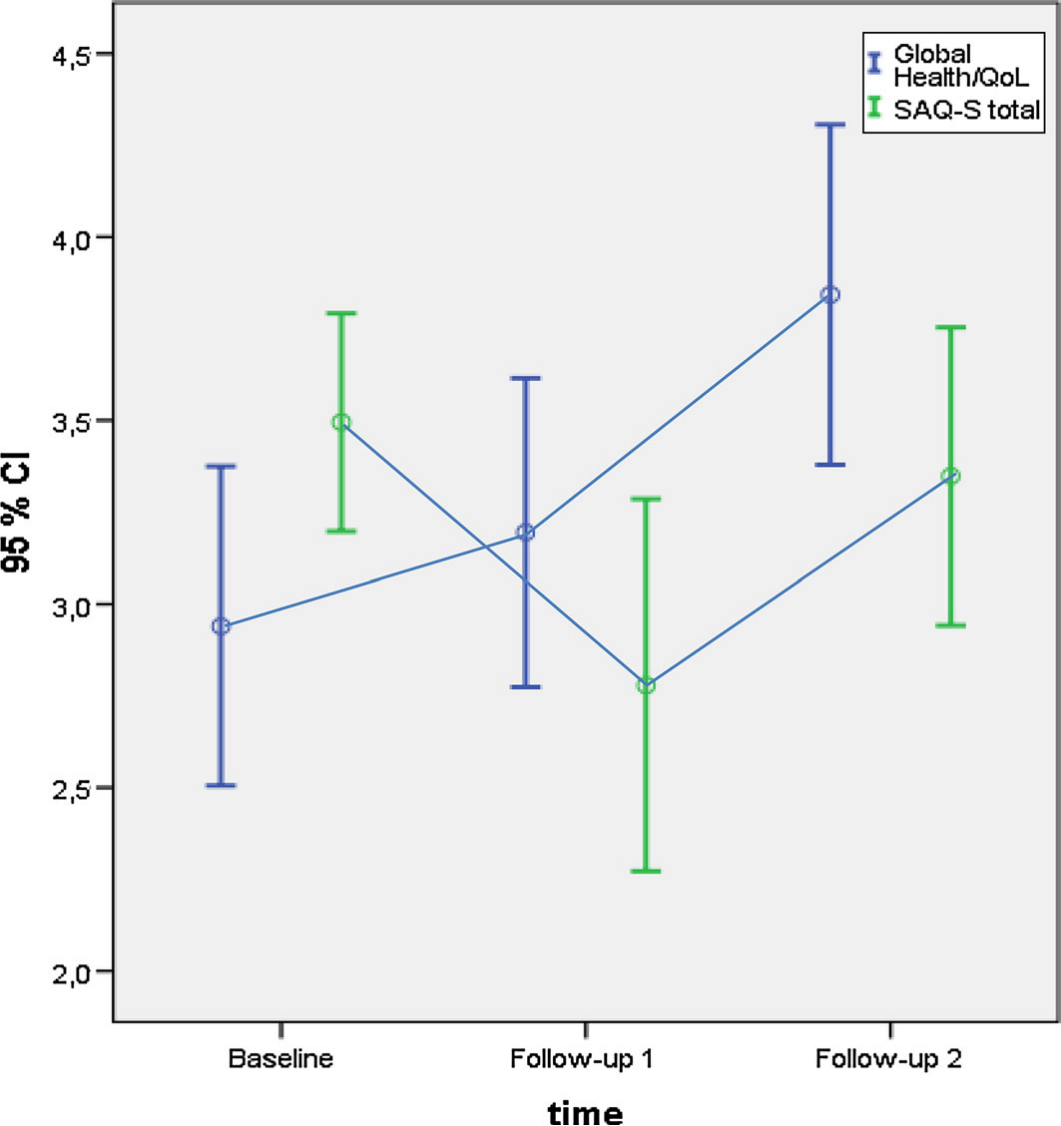
Mean values and confidence interval for *SAQ*-*S total* and *Global Health status*/*QoL Scale*, at Baseline, Follow-up 1 and Follow-up 2 (n = 18)

An overall description of changes during the study period based on total scores shows that the patients reported negatively affected sexuality (SAQ-S) at 1 month after treatment, compared to before treatment (Baseline: mean 3.49, CI 3.20–3.79, Follow-up 1: mean 2.78, CI 2.27–3.29). Six months after the treatment patients’ reported scores had almost returned to baseline (Follow-up 2: mean 3.35, CI 2.94–3.75). Regarding *Global Health status*/*QoL* a gradually improvement was reported during the occasions for follow-up with the lowest score reported at baseline (Baseline: mean 58.80, CI 50.10–67.50, Follow-up 1: mean 63.89, CI 55.48–72.30, and Follow-up 2: mean 76.85, CI 67.58–86.12). Body image was slightly affected during the follow-up period with the greatest impact reported 1 month after treatment (Follow-up 1: mean 12.22, CI 9.69–14.76, Follow-up 2: mean 11.00, CI 8.63–13.37). The changes related to SAQ-S and EORTC QLQ-C30 is illustrated in Fig. 2.

### Changes Between Baseline and Follow-up 1 Regarding SAQ-S and EORTC QLQ-C30

When comparing changes regarding sexuality (SAQ-S) on an item level most of the patients reported lower scores on a majority of the items at Follow-up 1 compared to Baseline. There was a statistically significant decrease in five of the unisex items scores. The patients reported decreased *desire for sexual activity*, *frequencies of sexual activity*, and *how often sexual activity was*/*is pleasurable.* They also more often *felt too tired for sexual activity*, and experienced worse *problems getting sexually aroused*/*turned on* (Table 2).

**Table 2:** Statistics for Sexual Adjustment Questionnaire, patients items score, baseline, Follow-up 1 and Follow-up 2

	Baseline (n = 20)		Follow-up 1 (n = 19)		Follow-up 2 (n = 20)		Baseline–Follow-up 1	Follow-up 1–Follow-up 2
	n	Mean (SD)	n	Mean (SD)	n	Mean (SD)	*P*	*P*
Sexual interest								
B. In the 6 months before you found out you had cancer, how important was sexual activity in your life?								
F. What is the importance of sexual activity in your life right now?	20	2.55 (.95)	19	2.21 (1.18)	19	2.68 (1.00)	.146	.172
B. Were you having sexual relations with anyone? (only baseline)	18	2.89 (1.03)						
B. Did you desire sexual activity?								
F. Do you have desire for sexual activity?	20	3.61 (.82)	19	2.79 (1.13)	20	3.35 (1.27)	.**005**	.**031**
B. Did you have a greater desire for sexual activity than your partner/partners?								
F. Do you have a greater desire for sexual activity than your partner/partners?	19	2.79 (1.03)	16	2.38 (1.41)	16	2.69 (1.30)	.188	.188
B. Was it you who initiated (started) sexual activity with your partner/s?								
F. Have you been the one to initiate sexual activity with your partner/partners since last cancer treatment?	20	3.00 (1.00)	15	2.47 (1.41)	14	2.86 (1.23)	.230	.766
B. Was it important for you to reach orgasm?								
F. Is it important for you to reach orgasm?	20	3.95 (1.05)	18	3.72 (1.23)	20	3.80 (1.20)	.547	.500
Sexual function								
B. Were you too tired for sexual activity?								
F. Do you feel too tired for sexual activity?	20	3.65 (.88)	18	3.06 (1.21)	19	3.37 (1.07)	.**044**	.371
B. Before finding out you had cancer, did you have problems getting sexually aroused/turned on?								
F. Do you have problems getting sexually aroused/turned on?	20	4.15 (1.14)	15	3.53 (1.46)	19	3.74 (1.33)	.**031**	.906
B. Did you have problems in reaching orgasm or did you feel that you came too soon?								
F. Since your cancer treatment, do you have problems in reaching orgasm or did you feel that you ‘come’ too soon?	20	3.80 (1.06)	11	4.27 (.79)	19	3.95 (1.13)	.656	1.00
B. Did you experience problems with your sexual ability before finding out you had cancer?								
F. Have you experienced problems with your sexual ability since your cancer treatment?	18	4.44 (.86)	12	3.50 (1.24)	18	4.00 (1.24)	.063	.133
Sexual satisfaction								
B. How often was sexual activity pleasurable?								
F. How often is sexual activity pleasurable for you now?	20	4.20 (.89)	16	3.31 (1.54)	19	3.68 (1.16)	.**016**	.234
B. How often did you have sexual activity (with or without a partner)?								
F. How often do you have sexual activity (with or without a partner)?	20	2.50 (.95)	19	1.32 (1.29)	20	2.00 (1.30)	.**004**	.**027**
B. Before finding out you had cancer, did you feel satisfied following sexual activity?								
F. Do you feel satisfied following sexual activity?	20	4.30 (.93)	12	4.08 (.90)	15	4.20 (1.08)	.500	.500
B. Were you satisfied with the frequency of sexual activity in your life?								
F. Are you satisfied with the frequency of sexual activity in your life?	20	3.70 (.98)	18	3.11 (1.18)	19	3.16 (1.21)	.074	.759
Items not included in a scale								
F. How soon after your last cancer treatment did you resume sexual activity (on your own or with another person)? (only follow up)			19	2.74 (1.79)	20	3.50 (1.47)		.077
B. Did you feel any pain or discomfort during sexual activity?								
F. Do you feel any pain or discomfort during sexual activity?	20	4.55 (.83)	11	4.45 (.93)	18	4.28 (1.13)	1.00	1.00
B. How has ‘having cancer’ changed your sexual relationship with your partner(s)?								
F. Has ‘having cancer’ changed your sexual relationship with your partner(s)?	17	2.35 (.79)	15	2.13 (.83)	16	2.31 (.70)	.688	.250
F. How has ‘undergoing cancer treatment’ changed your sexual relationship to your partner/partners?)? (only follow up)			15	2.20 (.78)	15	2.33 (.72)		1.00
B. Could you achieve an erection when sexually aroused?								
F. Can you achieve erection when sexually aroused? (male)	12	4.25 (.97)	10	3.90 (1.20)	12	4.17 (1.12)	.630	.688
B. Did you feel that it took you a long time to achieve an erection?								
F. Do you feel that it takes you long time to achieve an erection? (male)	13	3.85 (1.28)	9	3.44(1.51)	12	4.00 (1.04)	.810	.250
B. Did you experience vaginal dryness during sexual activity?								
F. Do you experience vaginal dryness during sexual activity? (female)	7	3.57 (1.40)	1	4.00	6	3.50 (1.52)		

Bold values indicate *P* ≤ .05

Responses range from 1 to 5 except for one item with a six point scale. Higher scores indicate more possible feelings or function in these areas

The response alternative 0 was used when the question was not applicable, except for the item with a six point scale

Wilcoxon signed rank test (exact sig. two-tailed) between baseline-Follow up 1, Follow up 1–Follow up 2

*B* Baseline, *F* Follow-up

Seven patients (37 %) out of nineteen reported ‘no importance’ of *sexual activity*, compared to baseline values (15 %, n = 3/20). The proportion of patients who scored ‘no problems’ *with sexual ability* decreased (baseline: 67 %: n = 12/18, Follow-up 1: 25 %, n = 3/12) indicating increasing problems. Nine patients (47 %) out of nineteen reported that they ‘had not yet’ *resumed sexual activity* 1 month after treatment and most patients (60 %, n = 9/15) reported *that undergoing cancer treatment had* negatively *changed the patients’ sexual relationship to partner*/*partners.*

When comparing changes in patients’ scores on an item and sub-scale level for the EORTC QLQ-30, no statistically significant differences were found (Table 3).

**Table 3:** Statistics of health related quality of life scores between the three occasions of measurement

Variables	HRQoL Baseline (n = 20)		HRQoL Follow-up 1 (n = 18)		HRQoL Follow-up 2 (n = 20)		Baseline–Follow up 1	Follow up 1–Follow up 2
	Mean	(SD)	Mean	(SD)	Mean	(SD)	*P*	*P*
Global health status/QoL^a^	60.00	(17.44)	63.89	(16.91)	75.42	(19.40)	.194	.**001**
Functional scales^a^								
Physical	69.67	(18.16)^c^	68.89	(21.81)	80.35	(18.52)	.561	<.**001**
Role	41.67	(29.37)	51.85	(34.25)	77.50	(28.24)	.511	.**001**
Emotional	78.75	(19.21)	81.48	(15.00)	82.92	(17.41)	.609	.505
Cognitive	80.83	(21.81)	86.11	(17.38)	84.17	(13.76)	.270	.453
Social	59.17	(27.29)	60.19	(23.67)	86.67	(18.42)	.965	<.**001**
Symptom scales/items^b^								
Fatigue	55.44	(25.97)	45.68	(20.43)	31.11	(20.90)	.406	.**002**
Nausea and vomiting	15.00	(21.56)	5.56	(16.17)	.83	(3.73)	.219	.250
Pain	16.67	(18.73)	20.37	(24.63)	21.67	(27.09)	.707	.999
Dyspnoea	33.33	(24.18)	38.89	(26.20)	18.33	(22.88)	.307	.**004**
Insomnia	35.00	(38.20)	18.52	(26.13)	31.67	(29.57)	.180	.234
Appetite loss	28.33	(27.09)	11.11	(22.87)	8.33	(18.34)	.072	.500
Constipation	18.33	(33.29)	9.26	(19.15)	5.00	(16.31)	.266	.500
Diarrhoea	15.00	(17.01)	7.41	(18.28)	6.67	(13.68)	.289	1.00
Financial difficulties	25.00	(33.98)	18.52	(30.73)	11.67	(19.57)	.438	.188

Bold values indicate *P* ≤ .05

Wilcoxon signed rank test (exact sig. two-tailed), between Baseline and Follow up 1, Follow up 1 and Follow up 2

^a^Score range from 0 to 100, with a higher score representing a higher level of functioning

^b^Score range from 0 to 100, with a higher score representing a greater degree of symptoms

^c^Baseline (n = 19)

### Changes Between Follow-up 1 and Follow-up 2 Regarding SAQ-S, BIS and EORTC QLQ-C30

When comparing changes regarding sexuality most patients reported higher scores at Follow-up 2 compared to Follow-up 1. There was a statistically significant increase in two of the unisex items: *desire for sexual activity*, as well as *frequencies of sexual activity* (Table 2). No other statistically significant differences were found.

At Follow-up 2, most patients (90 %, n = 17/19) reported that *sexual activity was of importance* in varying degree in their life at present, and the proportion of patients who scored ‘no problems’ regarding *problems with sexual ability* had increased (56 %, n = 10/18). Seventeen patients (85 %) out of 20 had *resumed sexual activity* or had ‘never stopped’ having sexual activity, while still more than half of patients (53 %, n = 8/15) reported that *undergoing cancer treatment had changed the patients’ sexual relationship to partner*/*partners.*

Regarding body image, patients reported statistically significant positive changes for two of the eight items: *feeling self*-*conscious about their appearance* and *feeling of less physically attractive as a result of your disease or treatment* (Table 4). In addition, at Follow-up 2: eight patients (44 %, n = 8/18) scored that Body image was ‘not at all’ affected (in all items).

**Table 4:** Statistics of Body Image Scales scores between the two occasions of Follow-up

Item	Follow-up 1 (n = 18)		Follow-up 2 (n = 18)		FU1–FU2
	Mean	(SD)	Mean	(SD)	*P*
Have you been feeling self-conscious about your appearance?	1.61	(.85)	1.22	(.55)	.**031**
Have you felt less physically attractive as a result of your disease or treatment?	2.28	(1.13)	1.67	(.77)	.**039**
Have you been dissatisfied with your appearance when dressed?	1.56	(.86)	1.28	(.58)	.125
Have you been feeling less feminine/masculine as a result of your disease or treatment?	1.39	(.70)	1.39	(.85)	1.00
Did you find it difficult to look at yourself naked?	1.28	(.75)	1.39	(.92)	.75
Have you been feeling less sexually attractive as a result of your disease or treatment?	1.67	(.91)	1.67	(.97)	1.00
Did you avoid people because of the way you felt about your appearance?	1.06	(.24)	1.06	(.24)	1.00
Have you felt dissatisfied with your body?	1.39	(.78)	1.33	(.84)	1.00

Bold values indicate *P* ≤ .05

Wilcoxon signed rank test (exact sig. two-tailed), between Follow up 1 and Follow up 2

Score range from 1 to 4, with a lower score representing a higher degree of body image

When comparing changes on an item and sub-scale level for the EORTC QLQ-C30 most of the patients reported higher scores at Follow-up 2 compared to Follow-up 1. The patients’ score for the scale *Global Health status*/*QoL* and in three of the five functional scales: *Physical Scale*, *Role Scale* and *Social Scale* showed a statistically significant increase. Regarding the three symptom scales and six single items, the patients reported less severity of symptoms and the scores were statistically significantly lower for the symptom item *fatigue* and *dyspnea.* No other differences were found (Table 3).

### Influence of Sexuality and Body Image on Changes in HRQoL

**Fig. 3:**
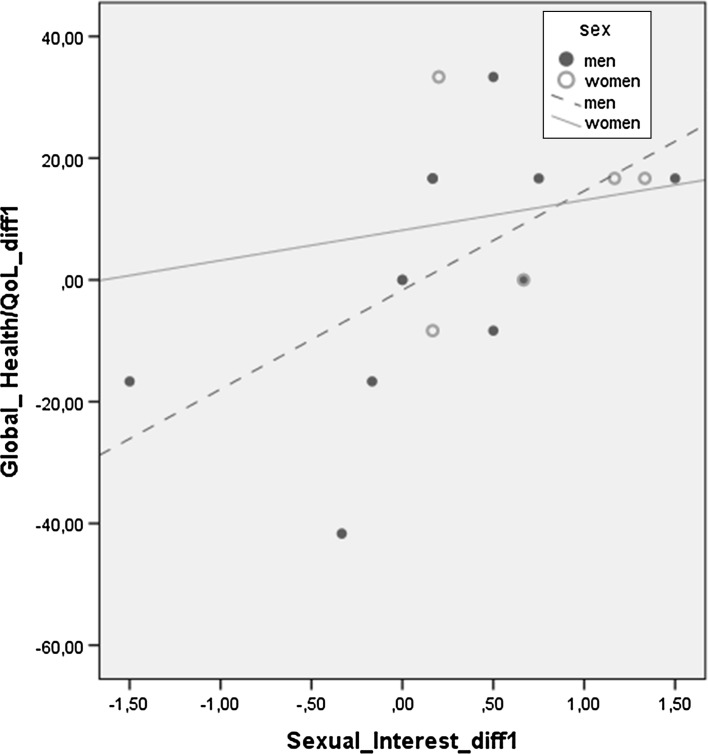
Linear regression: ‘changes in differences’ in *Global Health status*/*QoL* and *SAQ-S* *Sexual Interest * among men and women, between Baseline and Follow-up 1. Men R^2^ linear = .336, women R^2^ linear = .027

In the regression analysis, between Baseline and Follow-up 1 (*diff 1*), the results showed that changes in sexuality influenced changes in HRQoL. *The Sexual Interest diff 1* explained changes in *Global Health status*/*QoL diff 1* (B = 27.50, *P* = .009) (Table 5). Based on these results a further linear regression analysis was carried out which showed that *Sexual Interest diff 1* for men explained 33.6 % of the changes for *Global Health status*/*QoL diff 1.* The corresponding figure for women was 2.7 % (Fig. 3).

**Table 5:** Contribution of characteristic sex, SAQ-S dimensions diff and Body Image Scale diff on global health status/QoLdiff between the occasions of measurement

Global Health status/QoL-diff 1				Global Health status/QoL-diff 2			
	*B*	SE	*P*		B	SE	*P*
*Characteristics*				*Characteristics*			
Sex	−1.705	9.62	.862	Sex	−10.346	7.76	.210
*SAQ-S-scales*				*SAQ-S-scales*			
Sexual Interest diff 1	27.501	8.83	.**009**	Sexual Interest diff 2	−5.288	7.83	.513
Sexual Function diff 1	3.746	7.13	.609	Sexual Function diff 2	4.089	7.13	.578
Sexual Satisfaction diff 1	10.081	7.15	.184	Sexual Satisfaction diff 2	2.783	4.93	.584
				Body Image-diff 2	−2.211	.88	.**028**
R^2^	.484			R^2^	.488		
Adjusted R^2^	.312			Adjusted R^2^	.256		

Bold values indicate *P* ≤ .05

*diff* Differences in patients score between two occasions of measurement, *diff 1* between Baseline and Follow up 1, *diff 2* between Follow up 1 and Follow up 2

**Fig. 4:**
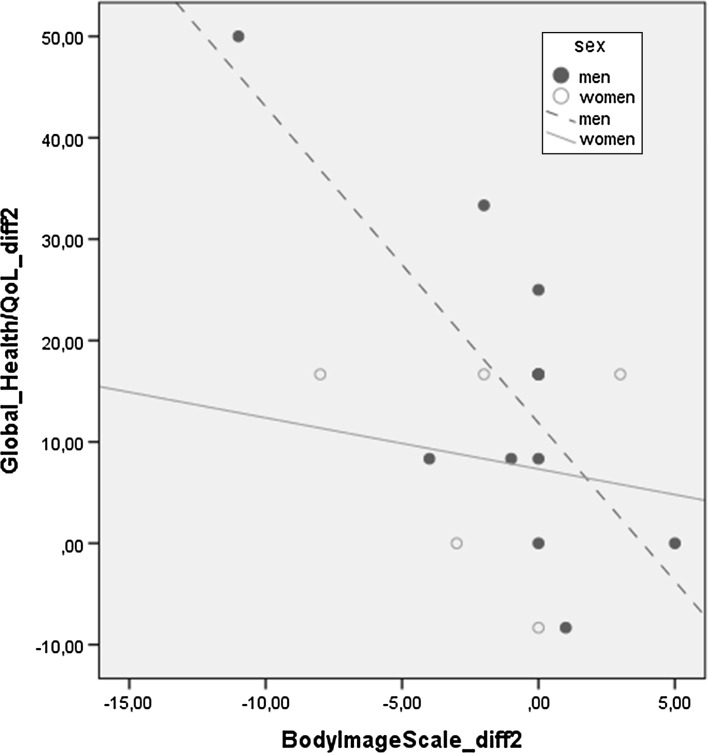
Linear regression: ‘changes in differences’ in *Global Health status*/*QoL* and *Body Image Scale* among men and women, between Follow-up 1 and Follow-up 2. Men R^2^ linear = .546, women R^2^ linear = .03

In the regression analysis, between Follow-up 1 and Follow-up 2 (*diff 2*), the results showed that changes in sexuality and body image influenced changes in HRQoL (Table 5). The *BIS diff 2* explained changes in *Global Health status*/*QoL diff 2* (B = −2.21, *P* = .028). A further linear regression analysis showed that *Body Image diff 2* for men explained 54.6 % of the changes for *global health status*/*QoL diff 2.* The corresponding figure for women was 3 % (Fig. 4).

## Discussion

This study provided data on self-estimated changes regarding sexuality, body image and HRQoL in 20 patient with hematologic malignancies treated with chemo- or chemoimmuno-therapy, above 45 years, from baseline until 6 months after treatment. The main results of this study showed that patients’ sexuality was negatively affected 1 month after treatment compared to baseline, and that after 6 months the patient reported scores had almost returned to baseline scores. Body image was slightly affected 1 month after treatment, and after 6 months 50 % of the patients reported that body image was not affected at all. Regarding HRQoL patients reported gradually improved scores during the study period and after 6 months the scores were comparable with a general population. Regression analysis showed that changes in sexuality and body image seemed to influence changes in HRQoL for men. The results from this study are an important contribution to the knowledge in this scarcely studied area, even though the sample size was small. This study provides both clinical relevant data in an area of importance for cancer rehabilitation and a direction to future research such as larger longitudinal studies in order to verify the result.

Body image is an important aspect of both sexuality [22, 37] and HRQoL [37]. In our study the patients reported that their body image was only slightly or not at all affected. Contradicting results are reported by Olsson et al. [22] who in a qualitative interview study found that patients treated for hematologic malignancies experienced an impact on their body image due to decreased physical and psychological strength. The experience of changed and unrecognized body was described as affecting the sexual relationship. Furthermore, Fallbjörk et al. [38] and Katz [39] conclude that satisfaction with one’s own body and the appearance are important factors in order to function sexually. Hence, the question about how to measure the impact of body image in an adequate way for this special group of patients remains to be answered in larger studies. Thereto, studies focusing supportive interventions related to body image and sexual relationships need to include evaluation of best ways of assessment and monitoring.

In this study the patients reported that HRQoL was negatively affected already at baseline. A probable reason could be that baseline data was collected at a point of time when side effects such as troublesome tiredness, insomnia and appetite loss often are severe. This must be taken into account when evaluating the findings. However, 1 month after treatment patients reported an improved HRQoL even though not statistically significant. It is noteworthy that non-statistically significant results could be of clinical importance [40]. Another way to judge changes regarding side-effects, symptoms and HRQoL could be to estimate clinical important differences [31]. Some researchers within the field of HRQoL argue that a difference between two occasions of measurement on a subscale ≥10-points reflects a difference of clinical importance [40, 41]. By using the ‘10-point difference method’ in our study, the functional scale *Role* and the symptom scales *fatigue*, *nausea and vomiting*, *insomnia* and *appetite loss*, showed improvements of clinical importance 1 month after treatment and after 6 months the HRQoL score was comparable to a general population [42]. In summary, our findings seem to be in line with earlier studies with participants in various ages showing that long-term cancer survivors regardless of age appear to recover almost completely with respect to physical, psychological and emotional well-being [3, 5].

In this study patients reported lack of sexual interest and fatigue was scored as the most distressing symptom. Although there is evidence regarding the impact of fatigue on physical, psychological and cognitive functioning [43] and global quality of life [44], its impact on sexuality in patients with hematologic malignancies is scarcely studied. The only study found showed that thoughts about sex simply were ‘not on the agenda’ due to a lack of physical and psychological strength [22]. This highlights the need to identify and assess fatigue and other symptoms affecting sexuality and design appropriate supportive interventions together with the patients and eventual partner.

The negative impact on patients’ sexual relationship due to cancer and its treatment described in this study has earlier been shown in studies on patients with sex-specific cancer [11, 45, 46]. In addition, in the case of existing relational problems before the cancer diagnosis, the problems have been shown to often worsen in the context of a cancer [45, 46]. Sexuality has in several studies been described as an unmet supportive care need [16, 47–49]. Support to the couples regarding affected sexual relationship could be assumed to be needed during the first 6 months after treatment when patients are resuming life again [22]. However, the patients in our study reported quite low importance of sexuality at all three occasions for measurement and similar findings has also been found in a qualitative study where patients also described few sexual problems and limited needs of support [22]. Sexuality is to a large extent seen as a private and sensitive topic by both patients and nurses [22, 50]. Therefore, we stress that it is a challenge to identify patients during treatment and cancer rehabilitation that ascribe sexuality importance and also want support regarding sexuality issues in order to avoid violation of patients’ integrity. Furthermore, the nursing education has to ensure that nurse student are trained in communication regarding sensitive and taboo issues such as sexuality. In addition, the health care leaders’ needs to acknowledge this area as being an important issue for nurses.

### Strengths and Limitations

Sexuality, especially in relation to body image and HRQoL, has rarely been studied in patients suffering from hematologic malignancies treated with chemo- or chemoimmuno-therapy. Therefore the results of this study are an important contribution to the body of knowledge in this area, even though the generalizability is limited due to the small size of the sample. The low response rates could be assumed to be due to the sensitive nature of the foci of the research [25], or because patient being exhausted due to severe symptoms and side effects of treatment [22].

Even if SAQ is not in total congruence with the WHO’s [51] definition of sexuality it was found to be the most suitable instrument for measuring affected sexuality in patients with hematologic malignancies. The other instruments found measuring affected sexuality were either sex-specific or considered to be too detailed regarding sexual dysfunction [52–54]. An instrument, which encompasses the physical, psychological, intellectual, and social aspects of sexuality, as outlined in the definition of sexuality by the WHO, would be preferable and an instrument where perspectives such as closeness, intimacy, tenderness and body image are included is warranted.

The inclusion of BIS, which assesses an individual’s feelings and attitudes to their body function and appearance, was an attempt to get closer to the WHO definition of sexuality. To our knowledge, BIS has not been used previously in studies about sexuality in patients treated for hematologic malignancies. However, the suitability of the content of the items in BIS for the study group needs to be discussed, as they mainly concern external appearance. Questions related to the internal experience of the body [55], which could be assumed to be of importance for patients treated for hematologic malignancies, are missing. Furthermore, two items were removed as they were considered to be applicable only to patients who have undergone surgery. The removed item, ‘have you been feeling that the treatment has left your body less whole’, might have given us additional information related to the internal experiences of affected body image.

A longitudinal design with repeated measurements was chosen in this study, in order to describe and explore changes in sexuality, body image and HRQoL from baseline until 6 months after treatment. For ethical reasons, baseline data were collected with connection to the second cycle of treatment, a time when side-effects often are severe. Therefore, recall bias has to be taken into consideration when evaluating the results. However, the questions in SAQ-S were designed as ‘how sexuality was experienced before diagnoses’ and can therefore be assumed to reflect the situation before diagnosis. Furthermore, since HRQoL has been described as already affected at the time for diagnosis in this group of patients due to symptoms of the disease, it is not possible to obtain a ‘true’ baseline regarding HRQoL.

The opportunity to further explore individual changes over time for the group of patients in this study is presented by the regression analysis, enabled by access to the modified SAQ scales. The sample size can be considered small for a regression analysis compared with the recommended ten cases of data for the predictor in the model [27] but the longitudinal design resulted in over 50 measuring points (17 patients on three occasions). The intention was not to create a model; rather the regression analyses were used to explore how sexuality and body image influenced HRQoL for the specific group of patients. These results cannot be generalized, but have interesting information which needs to be further studied in a larger sample.

### Conclusion and Implication for Practice

This longitudinal study has shown changes in sexuality, body image and HRQoL over time in patients above 45 years of age who were treated for hematologic malignancies. Sexuality and HRQoL became negatively affected, and body image was slight negatively affected after 1 month. The patients recovered to a great extent regarding all three areas within a period of 6 months. One notable finding, even though this study was small, was that sexuality and body image seemed to influence HRQoL for men at 1 and at 6 months. Hence, this study highlights the necessity of considering issues related to sexuality and body image in the supportive care of patients with hematologic malignancies. This might be most important during the first 6 months after treatment when patients adjust to the life as cancer survivors.

Assessment of problems and needs together with timely interventions such as information and support is valuable for patients both during and after treatment. This also applies for sensitive and taboo issues such as sexuality when preparing and adjusting to the life as cancer survivors. In the cancer rehabilitation team, the nurses, who are present during the entire trajectory of care, have a key role working with holistic individual nursing care aiming to promote well-being and as high quality of life as possible for the patients. However, when supporting the patients, it is important for nurses to timely recognize the diversity and complexity of the patients’ problems, including their unmet needs, in order to give adequate support. Therefore, the care should be provided within the framework of patient-centered care and organized in a way that implies continuity in the nurse-patient relationship. When needed, the nurse also initiates intervention from other professions of the rehabilitation team.

### Further Research

Longitudinal multicenter studies focusing how chemo- and chemoimmuno-therapy regimens affect patients’ sexuality, body image and HRQoL, enabling larger samples and follow-up throughout the rehabilitation period are needed for developing evidence based interventions. A larger sample is also needed in order to enable the evaluation and development of the SAQ-S and BIS for the use in studies with patient treated for hematologic malignancies. In addition, more knowledge is needed in order to understand how sexuality, body image and HRQoL relate and impact on each other.

Thereto, in order to identify the adequate way of practice, intervention studies focusing supportive care during treatment and the period of rehabilitation are needed from the perspectives of both patients and personnel. It would also be valuable to study the issue from the perspective of health care providers which could integrate issues related to health economy. Furthermore, in order to prevent a situation where cancer patients’ supportive care needs including sexuality is studied only from an outside perspective, members of patients associations ought to be included in the research team when planning study design and evaluation.

## References

[1] The National Board of Health and Welfare: Cancer Incidence in Sweden. Official Statistics of Sweden, Statistics-Health and Medical Care, Stockholm (2011). http://www.socialstyrelsen.se/Lists/Artikelkatalog/Attachments/18919/2012-12-19.pdf. Accessed 5 Jan 2014

[2] Long J, Versea L (2006). Treatment approaches and nursing considerations for non-Hodgkin’s lymphoma. Semin. Oncol. Nurs..

[3] Redaelli A, Stephens J, Brandt S (2004). Short- and long-term effects of acute myeloid leukemia on patients health related quality of life. Cancer Treat. Rev..

[4] Leak A, Mayer D, Smith S (2011). Quality of life domains among non-Hodgkin lymphoma survivors: an integrative. Leuk. Lymphoma.

[5] Persson L, Larsson G, Ohlsson O (2001). Acute leukaemia or highly malignant lymphoma patients’ quality of life over two years: a pilot study. Eur. J. Cancer Care..

[6] Bober S, Varela VS (2012). Sexuality in adult cancer survivors: challenges and intervention. J. Clin. Oncol..

[7] Cleary V, Hegarty J (2011). Understanding sexuality in women with gynaecological cancer. Eur. J. Oncol. Nurs..

[8] Lavin M, Hyde A (2006). Sexuality as an aspect of nursing care for women receiving chemotherapy for breast cancer in an Irish context. Eur. J. Oncol. Nurs..

[9] Katz A (2007). Breaking the Silence: On Cancer and Sexuality. A Handbook for Healthcare Providers.

[10] Tierney K (2008). Sexuality: a quality-of-life issue for cancer survivors. Semin. Oncol. Nurs..

[11] Galbraith ME, Crighton F (2008). Alterations of sexual function in men with cancer. Semin. Oncol. Nurs..

[12] Hughes M (2008). Alterations of sexual function in women with cancer. Semin. Oncol. Nurs..

[13] Kiserud C, Fosså A, Bjøro T (2009). Gonadal function in male patients after treatment for malignant lymphomas, with emphasis on chemotherapy. Br. J. Cancer.

[14] Arden-Close E, Eiser C, Pacey A (2011). Sexual functioning in male survivors of lymphoma: a systematic review. J. Sex. Med..

[15] Behringer K, Müller H, Görgen H (2013). Sexual quality of life in Hodgkin lymphoma: a longitudinal analysis by the German Hodgkin Study Group. Br. J. Cancer.

[16] Jonker-Pool G, Hoekstra H, van Imhoff G (2004). Male sexuality after cancer treatment—needs for information and support: testicular cancer compared to malignant lymphoma. Patient Educ. Couns..

[17] Thygesen KH, Schjødt I, Jarden M (2012). The impact of hematopoietic stem cells transplantation: a systematic review of the literature. Bone Marrow Transplant..

[18] Zittoun R, Suciu S, Watson M (1997). Quality of life in patients with acute myelogenous leukemia in prolonged first complete remission after bone marrow transplantation (allogeneic or autologous) or chemotherapy: a cross-sectional study of the EORTC-GIMEMA AML 8A trial. Bone Marrow Transplant..

[19] Beckjord EB, Arora NK, Bellizzi K, Hamilton AS, Rowland JH (2011). Sexual well-being among survivors of non-Hodgkin lymphoma. Oncol. Nurs. Forum.

[20] Kiserud C, Schover L, Dahl A (2009). Do male lymphoma survivors have impaired sexual function?. J. Clin. Oncol..

[21] Tierney K, Facione N, Padilla G (2007). Altered sexual health and quality of life in women prior to hematopoietic cell transplantation. Eur. J. Oncol. Nurs..

[22] Olsson C, Athlin E, Sandin-Bojö AK (2013). Sexuality is not a priority when disease and treatment side effects are severe: conceptions of patients with malignant blood diseases. J. Clin. Nurs..

[23] Weber C, Fliege H, Arck P, Kreuzer K-A, Rose M, Klapp B (2005). Patients with hematological malignancies show a restricted body image focusing on function and emotion. Eur. J. Cancer Care.

[24] Olsson C, Sandin-Bojö A-K, Bjuresäter K (2015). Patients treated for hematologic malignancies—affected sexuality and health related quality of life. Cancer Nurs..

[25] Waterhouse J, Metcalfe MC (1986). Development of the Sexual Adjustment Questionnaire. Oncol. Nurs. Forum..

[26] Bruner D, Scott C, McGowan D (1998). Validation of the sexual adjustment questionnaire (SAQ) in prostate cancer patients enrolled on Radiation Therapy Oncology Group (RTOG) studies 90-20 and 94-08. Int. J. Radiat. Oncol. Biol. Phys..

[27] Field A (2009). Discovering Statistics Using SPSS.

[28] Hopwood P, Fletcher I, Lee A, Al GhazalS (2001). A body image scale for use with cancer patients. Eur. J. Cancer.

[29] Stead M, Fountain J, Napp V, Garry R, Brown J (2004). Psychometric properties of the Body Image Scale in women with benign gynaecological conditions. Eur. J. Obstet. Gynecol. Reprod. Biol..

[30] Brislin R (1970). Back-translation for cross-culture research. J. Cross Cult. Psychol..

[31] Guillemin F, Bombardier C, Beaton C (1993). Cross-cultural adaption of health-related quality of life measures: literature review and proposed guidelines. J. Clin. Epidemiol..

[32] Aaronson NK, Ahmedzai S, Bergman B (1993). The European Organization for Research and Treatment of Cancer QLQ-C30: a quality-of-life instrument for use in international clinical trials in oncology. J. Natl. Cancer Inst..

[33] Bowling A (2001). Measuring Disease: A Review Of Disease-Specific Quality of Life Measurement Scales.

[34] Fayers PM, Aaronson N, Bjordal K (2001). The EORTC QLQ-C30 Scoring Manual.

[35] Northern Nurses’ Federation (2003). Ethical guidelines for nursing research in the Nordic countries. Nord. J. Nurs. Res. Clin. Stud..

[36] World Medical Association (WMA): WMA Declaration of Helsinki—Ethical Principles for Medical Research Involving Human Subjects. WMA, Helsinki (2013). www.wma.net/en/30publications/10policies/b3/index.html. Accessed 29 May 2015

[37] Hughes MK (2000). Sexuality and the cancer survivor. Cancer Nurs..

[38] Fallbjörk U, Karlsson S, Salander P (2010). Differences between women who have and have not undergone breast reconstruction after mastectomy due to breast cancer. Acta Oncol..

[39] Katz A (2007). Quality of life for men with prostate cancer. Cancer Nurs..

[40] Osoba D, Rodrigues G, Myles J (1998). Interpreting the significance of changes in health-related quality-of-life scores. J. Clin. Oncol..

[41] Oerlemans S, Mols F, Nijziel M (2011). The impact of treatment, socio-demographic and clinical characteristics on health-related quality of life among Hodgkin’s and non-Hodgkin’s lymphoma survivors: a systematic review. Ann. Hematol..

[42] Michelson H, Bolund C, Nilsson B (2000). Health-related quality of life measured by the EORTC QLQ-C30. Reference values from a large sample of the Swedish population. Acta Oncol..

[43] Glaus A, Crow R, Hammond S (1996). A qualitative study to explore the concept of fatigue/tiredness in cancer patients and in healthy individuals. Support. Care Cancer.

[44] Wettergren L, Björkholm M, Axdorph U, Langius-Eklöf A (2003). Individual quality of life in long-term survivors of Hodgkin’s lymphoma—a comparative study. Qual. Life Res..

[45] Holmberg SK, Scott LL, Alexy W, Fife B (2001). Relationship issues of women with breast cancer. Cancer Nurs..

[46] Maughan K, Heyman B, Matthews M (2002). In shadow of risk. How men cope with a partner’s gyunaecological cancer. Int. J. Nurs. Stud..

[47] Hall A, Lynagh M, Bryant J (2013). Supportive care needs of hematological cancer survivors: a critical review of the literature. Crit. Rev. Oncol. Hematol..

[48] Lobb EA, Joske D, Butow P (2009). When the safety net of treatment has been removed: patients’ unmet needs at the completion of treatment for hematological malignancies. Patient Educ. Couns..

[49] Molassiotis A, Wilson B, Blair S (2011). Unmet supportive care needs, psychological well-being and quality of life in patients living with multiple myeloma and their partners. Psycho-Oncology.

[50] Olsson C, Berglund A-L, Larsson M (2012). Patient’s sexuality—a neglected area of cancer nursing?. Eur. J. Oncol. Nurs..

[51] WHO (2006). Defining Sexual Health: Report of a Technical Consultation On Sexual Health, 28–31 January 2002.

[52] McCoy NL (2000). The McCoy Female Sexuality Questionnaire. Qual. Life Res..

[53] Rosen R, Brown C, Heiman J (2000). The female sexual function index (FSFI): a multidimensional self-report instrument for the assessment of female sexual function. J. Sex Marital Ther..

[54] Rosen R, Catania J, Pollack L (2004). Male sexual health questionnaire (MSHQ): scale development and psychometric validation. Urology.

[55] Merleau-Ponty, M.: Kroppens fenomenologi **(in Swedish)**. Phénoménologie de la perception **(in French)**. Daidalos, Gothenburg (1997)

